# Clinical efficacy of one-finger meditation massage on IBS-C based on the “gut-brain axis” theory: study protocol for a randomized controlled trial

**DOI:** 10.1186/s12906-023-04019-3

**Published:** 2023-06-06

**Authors:** Xiayang Zeng, Jingjing He, Xiaoyu Li, Peng Chen, Jinhong Zuo, Xinlei Cai, Zhenyu Fan, Jianpeng Qu

**Affiliations:** 1 Tui Na Department, Zhejiang Hospital, Hangzhou, China; 2 Zhejiang University of Traditional Chinese Medicine, Hangzhou, China; 3 Surgical Department, The Third Hospital of Zhejiang University of Traditional Chinese Medicine, Hangzhou, China

**Keywords:** IBS-C, Randomized controlled trial, One-finger meditation massage, Gut-brain axis

## Abstract

**Background:**

As a common disorder of the gastrointestinal tract, irritable bowel syndrome (IBS) can have negative effects on patients and society, with irritable bowel syndrome with constipation(IBS-C) accounting for a large proportion of these effects. The main clinical manifestations of IBS-C are constipation, abdominal pain, and abdominal distension, which seriously impact the quality of life of patients. The mechanisms of IBS are complex, and the gut-brain axis has been an emerging and recognized theoretical system in recent years. Based on the theory of the gut-brain axis and the theory of Chinese medicine, we designed this study to evaluate the efficacy of one-finger meditation massage in treating IBS-C.

**Methods/design:**

This is a randomized controlled trial. Eligible patients with irritable bowel syndrome (IBS-C) wererandomized 1:1 to a test group (massage plus probiotics) and a control group (probiotics). Patients in the test group weretreated once every 10 days for three consecutive courses of treatment (i.e., three months) and weregiven Bifidobacterium trifolium capsules 630 mg/dose three times daily 30 min after meals every day during the treatment period, with follow-up observations at the end of the third and sixth months of the treatment period. The control group weregiven Bifidobacterium trifolium capsules 630 mg/dose, 3 times a day for 3 months, with follow-up observations at the end of the third and sixth months of the treatment period. The primary outcome indicators are the concentrations of 5-HT and substance P and the IBS Severity Scale (IBS-SSS) assessment. Secondary outcomes are the Bristol Rating Scale (BRSA) score, the IBS Quality of Life Questionnaire (IBS-QOL scale) score, and the assessment of the effectiveness of the evidence. The results wereassessed at the pretreatment, posttreatment, and follow-up stages. Any side effects weresubject to assessment.

**Discussion:**

The aim of this trial is to provide a new method of treatment based on pharmacological treatment that is easy to use, easy to promote and has proven efficacy and to establish the efficacy and safety of treating IBS-C through this trial.

**Registration for Trial:**

Chinese Clinical Trial Registry ChiCTR2200066417 on 5 December 2022. https://www.chictr.org.cn/bin/project/edit?pid=183461

## Background

Irritable bowel syndrome (IBS) is a condition characterized by chronic recurrent abdominal pain, and functional bowel disorders with abnormal bowel movements or changes in bowel habits are some of the most common gastrointestinal disorders [1]. According to the definition, IBS lacks morphological changes and biochemical abnormalities that could explain the symptoms. It is currently a symptom-based diagnosis [2]. Over the past decade, with improved living standards, changing diets and lifestyles, and environmental changes, the number of consultations for this disease has been increasing every year. According to reported statistics, the prevalence of IBS from country to country worldwide is approximately 9–22% in Western countries, and epidemiological studies in Beijing and Guangzhou in China have found that 5.6% of the general population has IBS [3, 4]. In approximately two-thirds of people, IBS is related to their diet; most of them changed their diet, and these modifications caused them to have insufficient food [5, 6], which may be relevant to their gastrointestinal discomfort [6, 7]. Compared with healthy subjects, patients with IBS feel high levels of mental stress. Many patients are depressed, have sleep disturbances, and feel exhausted due to recurrent pain in the abdomen and changes in bowel habits, which lead to fears of serious illness. The overall rate of IBS with psychosomatic disorders is 40–90% [8]. In turn, symptoms of IBS are often exaggerated by stress and/or anxiety [9].

IBS-C is an IBS disease whose etiology and pathogenesis remain elusive. It is generally believed that gastrointestinal motility disorders [10, 11], visceral hypersensitivity [12], abnormal brain-gut axis regulation [13], inflammation of the mucous membranes [14], genetic predisposition to disease [15, 16], nutrition [17] and psychosocial stress [18] are closely related to the development of the disease process. Over the last few years, there has been increasing evidence that the symptoms of irritable bowel syndrome are linked to the regulation of the gut-brain axis [19–21]. A central player in the perpetuation of IBS symptoms is the integrated action and communication between the microbiota and the autonomic nervous system. This signaling pathway is recognized as the gut-brain axis (GBA). The GBA is a bidirectional axis of regulation, consisting of a neuroendocrine network that connects the nervous system between the gastrointestinal tract and the brain and acts as a mutual regulator. The microbiota has a crucial role in this communication [19, 22, 23]. Evidence suggests that immune responses from ecological dysbiosis of the microbiota can contribute to the gastrointestinal symptoms of IBS, which suggests that IBS is actually a disease of both the microbiota and GBA [19]. Brain-gut interactions occur through the neuro-endocrine network system and intestinal flora for gastrointestinal tract-brain interactions [24]. Neuroendocrine cells of the brain-intestinal axis are regulated by the enteric nervous system, autonomic nervous system, and central nervous system and maintain the normal operation of the brain-intestinal axis by secreting related neurotransmitters and hormones [25]. Long-term stimulation by various factors in the internal and external environment can lead to dysfunction of neuroendocrine immune regulation, causing a state of visceral sensory hypersensitivity and malfunction of gastrointestinal tract motility; as a result, abdominal discomfort, changes in bowel habits, pain, fullness, belching, nausea and other abnormalities of the brain and intestinal interaction can occur [26]. The whole process requires the participation of brain-gut peptides, which mainly regulate the gastrointestinal tract by transmitting information between the gastrointestinal nervous system, the central nervous system, and the effector cells of the gastrointestinal tract, thus completing the brain-gut interaction [27]. The main peptides are vasoactive peptide (VIP), neuropeptide Y (NPY), substance P (SP), neuro hypocretin (NT), calcitonin gene-related peptide (CGRP), and 5-hydroxytryptamine (5-HT). The pathophysiological mechanism of constipated IBS is currently considered to be visceral hypersensitivity. Among them, 5-hydroxytryptamine (5-HT) plays an important role in the increased visceral sensitivity of IBS [28]. 5-HT is stored in intestinal endocrine cells, which act on the nerves of the mucous membrane within the lamina propria, activating secretory motor responses and signaling intestinal conditions to the central nervous system by means of visceral afferents; its importance in the control of gastrointestinal function has been confirmed in both animal and clinical studies [29–32].

Irritable bowel syndrome is now classified according to the different clinical stool manifestations: constipation (IBS-C), diarrhea (IBS-D), mixed (IBS-M) or undefined (IBS-U) [33]. IBS-C is characterized by at least 25% of bowel movements of Bristol 1–2 (hard or lumpy stools) and less than 25% of defecating of Bristol 6–7 (loose or watery stools), with fewer than 3 defecations per week [34, 35]. Patients are usually type 1 or type 2 on the Bristol stool scale, with discomfort such as incomplete bowel movements, infrequent bowel movements, abdominal pain, and bloating. Statistically, approximately 35.6% of people with irritable bowel syndrome have a diagnosis of IBS-C, affecting 4.3-5.2% of American adults [36–38]. As a chronic disease, it is not life-threatening, but because of its prolonged symptoms, it has a serious impact on the quality of life of the patient, causes a decline in social functioning, increases the consumption of medical resources, and places a significant economic strain on the patient and society as a whole. Acting in accordance with the report, compared with healthy subjects, subjects with IBS symptoms have lower QOL [39, 40]. Furthermore, there have been reports that people with IBS-C often have reduced productivity due to experiencing distress and dysfunction [41, 42]. Statistically, in the US alone, IBS-C treatment estimates range from $1.3-1 billion per year, with annual indirect costs, mainly due to absenteeism and reduced productivity, ranging from $205 million to $20 billion [43]. Therefore, effective control of IBS-C is a health care issue of critical importance worldwide. However, current treatments for IBS-C currently target only a single symptom, and past studies have shown that treatments that focus only on bowel frequency, such as probiotics used to treat IBS-C that only enhance the frequency of bowel movements, have been relatively unsuccessful, with more than half of IBS-C patients reporting continued constipation [44, 45]. We therefore explored whether a therapy combined with pharmacotherapy could better treat IBS-C, not only by enhancing bowel motility but also by relieving other symptoms, such as abdominal pain and flatulence (bloating), and improving the quality of life and survival of patients, thus achieving greater social and economic benefits.

Massage therapy is one of the oldest forms of healing and has been in use for centuries in many civilizations and cultures, including China [46]. Massage therapy promotes blood and lymphatic fluid circulation, relieves muscles, removes the dross, and encourages body and mind relaxation [47, 48]. By stimulating the abdominal muscles and increasing abdominal pressure, abdominal massage promotes peristalsis of the gastrointestinal tract [49]. Abdominal massage has been reported to improve constipation by mechanically stimulating the associated neurologic/autonomic response [50–53]. Abdominal massage increases local and visceral circulation and decreases the time it takes for stool to pass by stimulating colonic motility [51–54]. One-finger meditation massage is a traditional Chinese medical massage therapy that stimulates the point lightly and is now widely used clinically for various diseases. One-finger meditation massage therapy has been used for over 200 years and is guided by the basic theories of Chinese medicine, such as the theory of yin and yang, five elements, meridians, and internal organs. The one-finger meditation pushing method has the unique advantages of a small contact area, powerful but not losing softness, deep penetration, and concentration. It has been reported that abdominal massage can treat insomnia, functional constipation, chronic gastritis, abdominal distension, and other diseases. In this paper, we describe a randomized controlled study on the IBS-C system and comprehensively and objectively observed and analyzed the clinical efficacy of one-finger meditation massage pushing abdominal acupuncture points in the treatment of IBS-C. Traditional Chinese medicine has a history of thousands of years, including Chinese massage. It has a unique theoretical system and traditional therapies have been shown to be effective in clinical trials and basic animal studies. Now we are trying to interpret our theories in a modern scientific way, and tens of thousands of clinical researchers have used modern medical theories to confirm the efficacy and safety of traditional therapies. In this study, we are trying to combine the theoretical system of the gut-brain axis with one-finger meditation massage for the first time. We are attempting to demonstrate that stimulation of acupuncture points using the one-finger meditation massage can regulate 5-HT and P substances and validate the theoretical system of the gut-brain axi. This approach has many advantages, such as being painless, economical, fast-acting, and providing significant long-term effects.

## Methods

### Objective

The overall objective of the study is the evaluation of the clinical rationality of the one-finger meditation massage of the abdomen for treating patients with irritable bowel syndrome (IBS-C), providing a basis for clinical promotion, to have better long-term efficacy compared with drug treatment alone, to take advantage of its painlessness and higher patient compliance, and to provide an exact and effective treatment for IBS-C in Chinese medicine.

### Trial design

**Fig. 1:**
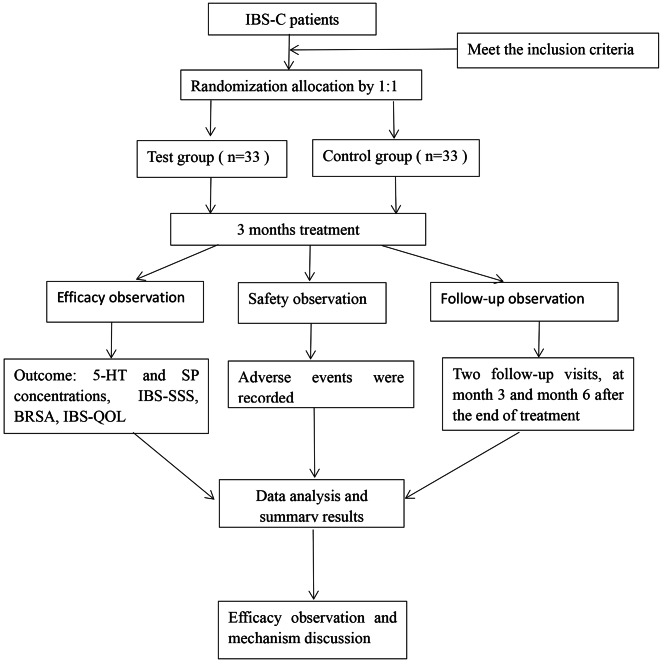
Flow chart of the study process. 5-HT, 5-hydroxytryptamine, SP, substance P, IBS-SSS, IBS Severity Scale, BRSA,Bristol Rating Scale Assessment, IBS-QOL, IBS Quality of Life Questionnaire

**Fig. 2:**
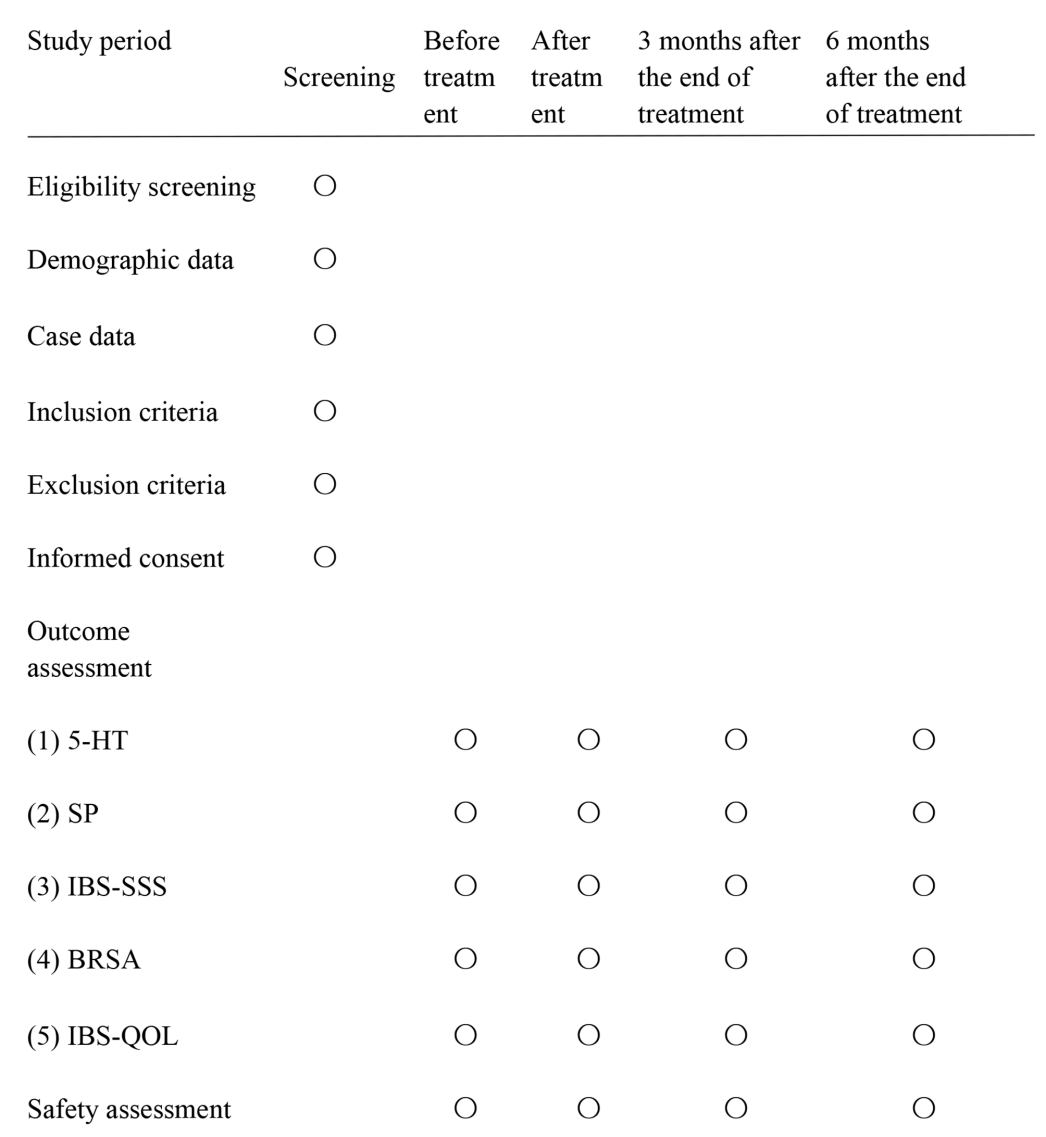
Schedule of enrolment, treatments, and assessments. O, required; 5-HT, 5-hydroxytryptamine; SP, Substance P; IBS-SSS, IBS Severity Scale (IBS-SSS) assessment; BRSA, Bristol Rating Scale Assessment; IBS-QOL, IBS Quality of Life Questionnaire

This trial is a single-center randomized, blinded trial in which outcome assessors were blinded to compare the efficacy of one-finger meditation massage combined with probiotic therapy versus pharmacotherapy in patients with IBS-C, which were conducted at Zhejiang Hospital. In total, 66 participants with IBS-C were randomly assigned to the trial and control groups. The protocol was registered on the following website: www.chictr.org.cn (No. ChiCTR2200066417). Protocol reporting is fully compliant with the SPIRIT guidelines [55]. Figure 1 shows the flowchart of the study process. In addition, the schedule for enrolling, treating and evaluating the trial is shown in Fig. 2.

### Participants, recruitment, and ethics

Recruitment of participants were from Zhejiang Hospital in two ways: recruitment posters and online recruitment. Participants were informed of the inclusion and exclusion criteria, interventions, intervention times, and other positive trial considerations during recruitment. Participants who meet the recruitment criteria will again be provided with the above information. After obtaining patient consent, patients will sign an informed consent form and be randomly assigned to a group. The Zhejiang Hospital Ethics Committee approved the study protocol (No. 2021 Pro-Audit No. 15 K).

### Diagnostic criteria

Refer to the < Rome IV Diagnostic Criteria for Functional Gastrointestinal Disorders>.

IBS typically presents with recurrent episodes of abdominal pain, at least 1 day per week for the last 3 months, associated with 2 or more of the following: (1) bowel movements; (2) changes in bowel frequency; and (3) changes in stool characteristics (appearance). IBS Constipation (IBS-C): At least 25% of bowel movements are Bristol 1–2, and less than 25% of bowel movements are Bristol 6–7.

### Inclusion criteria

Subjects with all of the following conditions were included: (1) meet the Western medicine diagnostic criteria of IBS-C; (2) aged between 20 and 80 years; (3) can adhere to the treatment as planned and can receive follow-up; (4) patients are conscious and can actively cooperate with the treatment, examination and complete the correct description of the assessment items; and (5) patients themselves or their relatives sign the informed consent.

### Exclusion criteria

Subjects with one of the following conditions were excluded: (1) those not meeting the above diagnostic and inclusion criteria; (2) pregnant or lactating patients; (3) patients with serious life-threatening primary diseases, including cardiovascular, cerebrovascular, hepatic, renal, hematopoietic and psychiatric diseases; and (4) those who receive other relevant treatments that may affect the observation of effect indicators in this study.

### Randomization and allocation concealment

An independent statistician who is not involved in the study will generate the randomization group: the numbers were randomly divided into two Groups A and B at a ratio of 1:1, i.e., A (Test group: Massage with the probiotic group) and B (Control group: probiotic group), using SPSS 19.0 software before the formal trial. The random sequence of the generated numbers and the grouping information were kept by the person in charge and kept confidential. Opaque brown envelopes were made according to the number of included cases, and the envelopes were numbered sequentially according to the order of numbers on the surface of the envelopes. The envelopes will contain the grouping information corresponding to the random sequence of numbers on the surface of the envelopes, such as 1(A) and 2(B). After signing the informed consent form and completing the basic information form, the envelopes were opened strictly in the order of the numbers on the envelopes, and patients were allocated to groups (trial or control) in accordance with the protocol in the envelopes.

### Blinding

This is a participant-rater-blinded study, meaning that participants do not know the group to which they belong. Participants have a 50% chance of being assigned to the test and control groups: massage with the probiotic group and probiotic group. The patient’s group allocation is also unknown to the follow-up assessors. Although the massage therapist will not be blinded to his or her treatment assignments, the massage therapist will not be part of the outcome assessment or data analysis.

### Intervention

#### Test group (massage with probiotic group)

The acupuncture points and areas are as follows: Zhongwan (RN12), Tianshu (ST25), Guanyuan (RN4), Qi Hai (RN6), Pishu (BL20), Shenshu (BL23), Dachangshu (BL25), Mingmen (DU4), Zusanli (ST36), Zhi Gou (SJ36) and abdomen. The locations of all selected acupoints are shown in Table 1. Operations: (1) Using the one-finger meditation massage method, the patient is in a supine position, and the operator uses the one-finger meditation massage method to push ST25, RN4, RN6, and RN12 for 2 min each; the one-finger meditation massage method requires concentration, sinking shoulder, dropping elbow, hanging wrist, palm deficiency, finger solid, tight pushing, and slow moving. The frequency of swinging is approximately 120–160 times/min; (2) Two minutes of clockwise abdominal massage; (3) Press and knead SJ36 and ST36 points for 1 min each; (4) The patient is in a prone position and rubs the lumbar back, BL23, DU4, BL20, and BL25 for approximately 2 min to penetrate the heat. The manipulation should be even, gentle, persistent, and powerful to achieve deep penetration; (5) Course of treatment: 10 days once, 3 times as a course of treatment, three consecutive courses of treatment (i.e., three months). The patient were given Bifidobacterium trifolium capsules (Shanghai Xinyi Pharmaceutical Co., Ltd., State Drug Administration S0950032) 630 mg/d, 3 times/d, 30 min after the meal for three months.

**Table 1:** Location of acupoints for treating IBS-C

Acupoints	Location
Zhongwan(RN12)	On the anterior median line, midpoint of the line connecting the lower end of the sternum and the belly button
Mingmen(DU4)	Interspinous process of the second and third lumbar vertebrae
Guanyuan(RN4)	In the lower abdomen, on the anterior median line, when the umbilicus is 3 cun below the middle.
QiHai(RN6)	In the lower abdomen. On the front median line, 1.5 cun below the middle of the umbilicus
Pishu(BL20, bilateral)	Under the spinous process of the 11th thoracic vertebra, open 1.5 cun laterally
Shenshu(BL23, bilateral)	Under the spinous process of the 2th lumbar vertebra, open 1.5 cun laterally
Dachangshu(BL25, bilateral)	Under the spinous process of the 4th lumbar vertebra, open 1.5 cun laterally
Tianshu(ST, bilateral)	In the abdomen, at the same level as the navel, 2 cun away from the front median line.
Zusanli(ST36, bilateral)	Three cun below Dubi (ST35), one finger -breadth (middle finger) from the anterior crest of tibia
ZhiGou(SJ36, bilateral)	On the dorsal side of the forearm, on the line between Yangchi and the tip of the elbow, 3 cun above the transverse wrist line, between the ulna and the radius

### Control group (probiotic group)

The patients were given Bifidobacterium trifolium capsules (Shanghai Xinyi Pharmaceutical Co., Ltd., State Drug Administration S0950032) 630 mg/d, 3 times/d, 30 min after the meal; the course of treatment were three months. Patients were advised to cultivate good lifestyle habits, avoid dairy products, soybeans, and other gas-producing foods and encouraged to consume moderate amounts of high-fiber foods.

### Outcome measures

#### Primary outcome

##### 5-HT and SP concentration

Serum 5-HT and SP concentrations in patients were determined before and after treatment using an enzyme-linked immunosorbent assay (ELISA). The level of 5-HT is closely related to the presence of diarrhea or constipation in irritable bowel syndrome, as well as SP [55, 56]. We will compare the efficacy of the two groups by ELISA of serum 5-HT and SP in patients before and after treatment.

##### IBS Severity Scale (IBS-SSS) assessment

Outcomes included the level of abdominal pain, frequency of abdominal pain, level of bloating, bowel satisfaction and effects on life. (1) Abdominal pain severity, frequency of pain in the abdomen, abdominal distension degree, defecation satisfaction, and effects on life were included. (2) Abdominal pain frequency score: abdominal pain frequency score = actual abdominal pain days/14 × 100. (3) IBS-SSS scale total score: IBS-SSS scale total score = abdominal pain severity score + abdominal pain frequency score. IBS-SSS scale total score = abdominal pain score + abdominal pain frequency score + abdominal distension score + bowel satisfaction score + impact on life satisfaction score + impact on life score.

#### Secondary outcome

##### Bristol rating scale assessment

Used to assess stool properties under visual observation during bowel preparation. 1 point: scattered hard masses, resembling nuts; 2 points: similar to salami but lumpy; 3 points: similar to salami but with superficial cracks; 4 points: similar to salami or snake, smooth and supple; 5 points: supple masses, with clear edges; 6 points: fluffy material, with indistinct edges, paste-like stool; 7 points: watery, without solids.

##### IBS Quality of Life Questionnaire (IBS-QOL scale)

The total score of the scale and the score of each dimension of the scale were calculated separately. The scale consists of 8 dimensions: poor mood, behavioral disorders, self-image, health concerns, avoidance of food, social functioning, sexual behavior and expansion of relationships. The total scale score and each dimension score were converted into standard scores according to the following formula. Standard score = (actual score sum of each item - theoretical minimum score/theoretical score range) ×100%.

##### Evaluation of the evidence efficacy

In accordance with the Guiding Principles for Clinical Research on New Chinese Medicines (Trial), the efficacy index was calculated using the nimodipine method: efficacy index = [(pretreatment score - posttreatment score)/pretreatment score] × 100%, divided into four levels: clinically cured, effective, and ineffective. (1) Clinically cured: disappearance or almost disappearance of major symptoms and signs, effectiveness ≥ 95%; (2) effective: a significant improvement in the main symptoms and signs, 70% ≤ effectiveness < 95%; (3) a significant improvement in the main symptoms and signs, 30% ≤ effectiveness < 70%; (4) ineffective: no significant improvement in the main symptoms and signs, 30% ≤ effectiveness < 95%;

Follow-up observation: (1) Follow-up and observation of all healed and effective cases; (2) Follow-up and re-evaluation every 3 months for a total of 2 times; (3) Follow-up can be done by telephone or letting the patient come to the hospital for re-evaluation or visiting the hospital if necessary; (4) Symptom re-evaluation with a score ≥ 1 level is determined as a relapse, and the follow-up will be stopped next time.

#### Statistical methods

##### Sample size

The test group sample size (massage with the probiotics group) and the control group (probiotics group) were randomly grouped in a 1:1 ratio, and the sample size was determined according to the bilateral hypothesis test, setting the test criterion as a = 0.05 (bilateral) and test efficacy as β = 0.20. The efficiency of the test group in the preexperimental observation is 90, and the efficiency of the control group is 60. The required sample was calculated to be approximately 60 cases, with an increase of 10% of cases taken off, and the actual sample size is approximately 66 cases; that is, 33 cases in the test group and 33 cases in the control group are selected.

### Statistical analysis

Two independent researchers will record all experimental data on CRF forms and enter them into the computer. Data will counted by statisticians using SPSS 19.0 statistical software. The mean ± standard deviation (M ± SD) will used for measurement data, paired sample tests will be used for before-and-after control within groups, independent sample tests will used for comparisons between groups, and chi-square tests will be used for efficacy grading. p < 0.05 is considered a statistically significant difference.

## Discussion

The complex and multifactorial nature of the pathogenesis of IBS means that single-drug treatments, such as probiotics, prebiotics, antimicrobials, anti-anxiety and antidepressant treatments and fecal transplants, are not as effective overall [56]. Complementary alternative medicine, including massage therapy, has been reported to be increasingly popular among clinicians and patients [57, 58].

Massage treatment has shown the advantages of safety, simplicity, economy, and nontoxic side effects. According to the study, one-finger meditation massage stimulates acupuncture points through gentle manipulation and thus exerts the therapeutic effect of the points. It is characterized by the continuous alternation of the force characteristics of the double waveforms during operation [59]. Using the fingers instead of acupuncture needles to stimulate the acupuncture points produces an effect similar to acupuncture, but compared to acupuncture, the one-finger acupressure technique has the advantages of noninvasiveness and safety. In our study, we focused on abdominal tui-na, using the technique to promote intestinal peristalsis on the one hand and selecting relevant acupuncture points to play the role of acupuncture points on the other hand.

Although massage treatment of irritable bowel syndrome has been proven effective, since IBS-C clinical research is still in its infancy, there are great shortcomings, and the number of clinical trials is very small, mostly relying on personal experience reports. Furthermore, there is a lack of evidence-based treatment and randomized controlled studies, poor credibility of the efficacy, and insufficient mechanism of research evidence to guide clinical practice. In our study, we conducted a randomized controlled study of massage treatment of IBS-C, which is the first of its kind in clinical practice, using the gut-brain axis as the theoretical basis. In addition to traditional abdominal tuina manipulation treatment, we combined traditional medicine acupressure therapy with the use of one-finger meditation therapy. The results of this study will continue to provide new ideas and methods for the treatment of irritable bowel syndrome-related conditions with massage. Ultimately, our hope is that the results of this trial will alleviate the symptoms of IBS-C patients, reduce their physical and psychological stress and improve their quality of life.

## Data Availability

This trial has been audited by the China Clinical Trials Registry and the experimental data will be shared on the Public Management Platform for Clinical Trials, the website is http://www.medresman.org.cn.
